# Supplementary material for: Human G-MDSCs are neutrophils at distinct maturation stages promoting tumor growth in breast cancer
Source: Life Sci Alliance. 2020 Sep 21;3(11):e202000893. doi: 10.26508/lsa.202000893 (PMC7536824; doi:10.26508/lsa.202000893)
Supplement: Supplementary file 2 [file LSA-2020-00893_TableS2.docx]

**Supplementary Table 2.** Antibodies used for flow cytometry (specificity; clone; dilution; distributor)

| anti-CD123-FITC (human, clone # 7G3, 1:20, BD Biosciences) |
| --- |
| anti-CD33-APC (human, clone # WM-53, 1:10, BD Biosciences) |
| anti-CD66b-PE (human, clone # G10F5, 1:20, BD Biosciences) |
| anti-CD14-PerCP (human, clone # 61D3, 1:20, Thermo Fisher) |
| anti-CD11c-PeCy7 (human, clone # B-ly6, 1:20, BD Biosciences) |
| anti-CD34-APC (human, clone # 581, 1:20, BD Biosciences) |
| anti-CD15-PE (human, clone # HI98, 1:20, BD Biosciences) |
| anti-CD11b-PE (human, clone # ICRF44, 1:20, BD Biosciences) |
| anti-HLA-DR-FITC (human, clone # L243, 1:25, BD Biosciences) |
| anti-CD127-Biotin (human, clone # HIL-7R-M21, 1:20, BD Biosciences) |
| anti-CD90-PE (human, clone # 5E10, 1:100, BD Biosciences) |
| anti-CD64-PE (human, clone # 10.1, 1:20, BD Biosciences) |
| anti-CD45-FITC (human, clone # HI30, 1:10, BD Biosciences) |
| anti-Collagen I-Biotin (human and mouse, cat #600-406-103, 1:20, Rockland) |
| anti-α-SMA-APC (human, mouse, and rat, clone # 1A4, 1:20, R&D Systems) |
| anti-CD33-FITC (human, clone # HIM3-4, 1:10, BD Biosciences) |
| anti-CD14-FITC (human, clone # M5E2, 1:10, BD Biosciences) |
