# Supplementary material for: Human G-MDSCs are neutrophils at distinct maturation stages promoting tumor growth in breast cancer
Source: Life Sci Alliance. 2020 Sep 21;3(11):e202000893. doi: 10.26508/lsa.202000893 (PMC7536824; doi:10.26508/lsa.202000893)
Supplement: Supplementary file 3 [file LSA-2020-00893_TableS3.docx]

**Supplementary Table 3.** Mass cytometry antibodies and labels

|  | Metal tag | Ab/beads/code | Clone |
| --- | --- | --- | --- |
| 1 | Y89 | CD45 | Hl30 |
| 2 | Pd102 | Barcode | Na |
| 3 | Pd104 | Barcode | Na |
| 4 | Pd105 | Barcode | Na |
| 5 | Pd106 | Barcode | Na |
| 6 | Pd108 | Barcode | Na |
| 7 | Pd110 | Barcode | Na |
| 8 | Ce140 | EQ Beads | Na |
| 9 | Pr141 | CD47 | B6H12 |
| 10 | Nd142 | CD19 | HIB19 |
| 11 | Ce142 | EQ Beads | Na |
| 12 | Nd143 | Ckit | 104D2 |
| 13 | Nd144 | CD31 | WM59 |
| 14 | Nd146 | CD64 | 10.1 |
| 15 | Sm147 | CD13 | WM15 |
| 16 | Sm149 | CD127 | A019D5 |
| 17 | Eu151 | CD123 | 6H6 |
|  | Eu151 | EQ Beads | Na |
| 18 | Eu153 | CD7 | CD7-6B7 |
|  | Eu153 | EQ Beads | Na |
| 19 | Sm154 | CD3 | UCHT1 |
| 20 | Gd155 | PD-1 | EH12.2H7 |
| 21 | Gd156 | CD10 | HI10a |
| 22 | Tb159 | CD11c | Bu15 |
| 23 | Gd160 | CD14 | M5E2 |
| 24 | Dy161 | CD90 | 5E10 |
| 25 | Dy162 | CD133 | AC133 |
| 26 | Dy163 | CD105 | 43A3 |
| 27 | Ho165 | CD16 | 3G8 |
|  | Ho165 | EQ Beads | Na |
| 28 | Er166 | CD34 | 581 |
| 29 | Er167 | CD38 | HIT2 |
| 30 | Gd169 | CD33 | WM33 |
| 31 | Er170 | CD45RA | HI100 |
| 32 | Yb171 | CD66a | CD66a-B1.1 |
| 33 | Yb172 | CD15 | W6D3 |
| 34 | Yb174 | HLA-DR | L243 |
| 35 | Lu175 | CD71 | OKT-9 |
|  | Lu175 | EQ Beads | Na |
| 36 | Yb176 | CD56 | NCAM16.2 |
| 37 | Ir191 | Intercalator | Na |
| 38 | Ir193 | Intercalator | Na |
| 39 | Bi209 | CD11b | ICRF44 |
