# Supplementary material for: Human G-MDSCs are neutrophils at distinct maturation stages promoting tumor growth in breast cancer
Source: Life Sci Alliance. 2020 Sep 21;3(11):e202000893. doi: 10.26508/lsa.202000893 (PMC7536824; doi:10.26508/lsa.202000893)
Supplement: Supplementary file 4 [file LSA-2020-00893_TableS4.docx]

**Supplementary Table 4.** Primers used for RT-qPCR

mCX3CL1 F: CTCACGAATCCCAGTGGCTT

mCX3CL1 R: GCAAGGTCTTCCAATGTGGC

mLY6C F: CTCAGGGACTGCAGTGCTAC

mLY6C R: AAAGAAAGGCACTGACGGGT

mGADPH F: TGCACCACCAACTGCTTAG mGADPH R: GGATGCAGGGATGATGTTC

mHPRT F: TGACACTGGCAAAACAATGCA mHPRT R: GGTCCTTTTCACCAGCAAG
