# Supplementary material for: Human G-MDSCs are neutrophils at distinct maturation stages promoting tumor growth in breast cancer
Source: Life Sci Alliance. 2020 Sep 21;3(11):e202000893. doi: 10.26508/lsa.202000893 (PMC7536824; doi:10.26508/lsa.202000893)
Supplement: Supplementary file 5 [file LSA-2020-00893_TableS5.docx]

**Supplementary Table 5.** Antibodies used for immunohistochemistry (specificity; clone; dilution; distributor)

anti-CD11b (human; clone #EP1345Y; 1:100; Abcam)

anti-α-SMA (mouse and human; clone #1A4; 1:1000; Agilent)

anti-human S100A9 (human; calgranulin B clone #H90; 1:2000; Santa Cruz Biotechnology)

anti-CD68 (human; clone KP1; 1:1500; Agilent)

anti-CD34 (mouse; clone #MEC14.7; 1:800; Santa Cruz Biotechnology)

anti-CD163 (human; clone #10D6; 1:250; Novocastra)

anti-CD31 (human; clone #JC70A; 1:200; Agilent)

anti-CD31(mouse and rat; cat #AF3628; 1:100; R&D Systems)

anti-Lyve-1 (mouse and human; clone #14917; 1:500; Abcam)

anti-Ki67 (human; clone #MIB-1; 1:200; Agilent)

anti-CD15 (human; clone Ab754; 1:100; Abcam)

anti-MPO (human; clone A0398; 1:250; Agilent)

anti-Ly6C (mouse; clone ER-MP20 (Ab15627); 1:100; Abcam)

anti-Ly6G (mouse; clone RB6-8C5; 1:250; LSBio)

anti-3-nitrotyrosine (mouse and human; clone 39B6; 1:100; Santa Cruz Biotechnology)

anti-PDGFRβ (mouse and human; clone 28E1; 1:100; Cell Signaling)

anti-F4/80 (mouse; clone BM8; 1:50; eBioscience)
